# Algorithmic short-video music consumption and academic related functions: a three-wave longitudinal study of university students

**DOI:** 10.3389/fpsyg.2026.1907189

**Published:** 2026-08-27

**Authors:** Jia Dong, Hailong Zhang, Jianjian Zhao, Ruikai Yuan

**Affiliations:** 1 Nanjing Normal University, Nanjing, China; 2 Linzhou College of Architectural Technology, Linzhou, China; 3 Anshan Vocational and Technical College, Anshan, China; 4 Southeast Univeristy, Nanjing, China

**Keywords:** academic flow, academic procrastination, attentional control, Chinese, university students, cross-lagged panel model, short-video music consumption

## Abstract

**Introduction:**

Short-video platforms have become deeply embedded in Chinese university students’ daily routines, and the music delivered through algorithmically curated short clips differs in important ways from traditional, self-selected music listening.

**Methods:**

The present study tested whether algorithm-driven short-video music consumption is prospectively associated with attentional control, academic procrastination, and academic flow across one academic term. A three-wave longitudinal survey was conducted across one academic term, with measurement intervals of approximately 6 weeks. Chinese university students (*N* = 920 at Time 1; *N* = 820 retained at Time 3) completed measures of short-video music consumption, attentional control, academic procrastination, and academic flow at each wave. An observed-variable cross-lagged panel model was estimated to examine autoregressive stability and hypothesized cross-lagged paths, with reverse paths and baseline daily short-video use included for robustness.

**Results:**

The results indicated that higher short-video music consumption prospectively predicted lower attentional control across both intervals (*β*s = −0.17 and −0.12); better attentional control predicted lower subsequent academic procrastination (*β*s = −0.23 and −0.14) and higher subsequent academic flow (*β*s = 0.18 and 0.14); and higher academic procrastination predicted lower subsequent academic flow (*β*s = −0.20 and −0.14). Bootstrapped indirect effects from short-video music consumption to academic procrastination (*β*s = 0.024) and academic flow (*β*s = −0.024) through attentional control were significant, as was the indirect effect from attentional control to academic flow through procrastination (*β*s = 0.031).

**Discussion:**

The findings highlight the value of distinguishing intentional music listening from algorithmically delivered short-video music consumption when considering students’ academic self-regulation in contemporary digital media environments.

## 1 Introduction

The daily lives of contemporary university students unfold within an increasingly algorithm-mediated digital media ecology, in which short-video platforms occupy a central role in leisure, social interaction, and emotional regulation. In China, platforms such as Douyin, Kuaishou, Bilibili short videos, and Xiaohongshu have become deeply embedded in students’ everyday routines, capturing substantial portions of attention that students previously allocated to other activities (Montag et al., 2021; Mu et al., 2022; Ye et al., 2022; Qu et al., 2024; Yang 2024). The proliferation of these platforms is not only a media-consumption phenomenon but also a psychological and educational concern: by structuring how attention is captured, sustained, and shifted across small fragments of content, algorithm-driven short-video environments may reshape the habits of attention regulation and learning rhythm that underpin academic functioning (Lu et al., 2022; Yan et al., 2024). As university students increasingly move between demanding academic tasks and algorithmically recommended short-form content, sustained attention and deep learning engagement may become more difficult to maintain (Chen et al., 2023b; Jiang et al., 2024).

Within this short-video ecology, music plays a distinctive yet under-examined role. Music in short-video platforms differs in important ways from the music engagement examined in much of music psychology. Traditional music listening is typically intentional, continuous, and self-selected: listeners choose tracks for specific purposes, allow them to unfold over time, and integrate them into self-regulated activities such as study, exercise, or relaxation (Saarikallio, 2011; Schäfer et al., 2013; Juslin, 2013). By contrast, the music encountered through short-video platforms is fragmented, algorithmically curated, emotionally amplified, and tightly bound to visual stimulation, social trends, and platform-driven retention mechanisms (Huang et al., 2022; Wang and Wang, 2024). Background tracks, trending songs, and recommended music-based clips can heighten emotional salience, encourage repeated viewing, and prolong platform engagement well beyond the duration users initially intended (Lu et al., 2022; Mu et al., 2022). The present study therefore reframes the construct of interest: it is not music listening in the conventional sense, but short-video music consumption within algorithmic platforms, which combines musical engagement with platform-driven attentional capture.

Among the psychological capacities potentially affected by such consumption, attentional control occupies a central position. Attentional control refers to the capacity to sustain task focus, resist distraction, shift attention flexibly when required, and return to ongoing tasks after interruption (Derryberry and Reed, 2002; Eysenck et al., 2007; Petersen and Posner, 2012). Repeated exposure to short, emotionally salient, and algorithmically sequenced music-based clips may gradually train students into patterns of attention fragmentation (Tan et al., 2024; Ophir et al., 2009; Parry and le Roux, 2021; Yan et al., 2024). Attention fragmentation refers to the theoretical phenomenon of attention being repeatedly diverted and recaptured by external stimuli in algorithmic media environments, whereas attentional control denotes the measured construct, scored such that lower scores reflect greater susceptibility to such fragmentation. Algorithmically driven short-video music consumption, by this account, does not simply consume time; it may also alter how students allocate and sustain attention in everyday learning contexts (Ye et al., 2025; Chen et al., 2023b).

Attentional control, in turn, has been linked to academic self-regulation. Academic procrastination is commonly defined as the voluntary delay of academic tasks despite the expectation of negative consequences (Steel, 2007; Steel and Klingsieck, 2016). Procrastination is increasingly understood not merely as a problem of time management or motivation, but as a self-regulatory difficulty that draws heavily on attentional capacities (Baumeister and Heatherton, 1996; Sirois and Pychyl, 2013; Zhao and Kou, 2024): students must initiate effortful tasks, resist competing digital cues, and sustain engagement under conditions of immediate reward asymmetry between learning tasks and short-video content (Hofmann et al., 2012; Tice and Bratslavsky, 2000; Liu et al., 2025). When attentional control is weaker, the threshold for switching away from demanding academic work toward immediately rewarding stimuli is lowered, and the likelihood of voluntary delay is correspondingly increased (Xie et al., 2023; Liu et al., 2022). Lower attentional control may therefore prospectively elevate academic procrastination across an academic term.

A further consequence of compromised attention and self-regulation is reduced academic flow. Academic flow describes a state of deep absorption, concentration, and intrinsic involvement in academic tasks, supported by sustained attention and a balance between perceived challenge and skill (Csikszentmihalyi, 1990; Engeser and Rheinberg, 2008; Shernoff et al., 2003; Leipold et al., 2025). Because flow depends on continuous and uninterrupted engagement, both lower attentional control and higher academic procrastination may undermine the conditions required for its emergence: lower attentional control reduces the immersive sustained focus that flow demands, while procrastination delays task entry and generates time pressure, guilt, and fragmented work patterns that interrupt the flow process (Steel, 2007; Sirois and Pychyl, 2013). Attentional control may thus relate to academic flow both indirectly, through academic procrastination, and directly, by enabling continuous task immersion.

Despite an accumulating literature on each of these constructs individually, three gaps remain. First, existing research on short-video use among university students has focused primarily on screen time, problematic social media use, and smartphone addiction, with comparatively little attention to the music-mediated mechanisms through which short-video platforms capture and retain attention (Shannon et al., 2022; Ansari et al., 2024; Zhang et al., 2020; Chen et al., 2023a; Zhang et al., 2024). Second, music psychology has traditionally treated music as a positive emotional or cognitive resource, yet far less is known about how music functions when embedded in algorithm-driven short-video environments, where listening is rapid, fragmented, and tied to algorithmic recommendation (Saarikallio, 2011; Schäfer et al., 2013; Wang and Wang, 2024). Third, much of the existing evidence relies on cross-sectional designs, which cannot adequately disentangle the temporal ordering among media consumption, attentional control, self-regulation, and learning experience (Hamaker et al., 2015; Lucas, 2023). What remains unclear is whether algorithm-driven short-video music consumption is prospectively associated with subsequent changes in attentional control, academic procrastination, and academic flow across an academic term.

To address these gaps, the present study employed a three-wave longitudinal design among Chinese university students to examine prospective associations among short-video music consumption, attentional control, academic procrastination, and academic flow. The choice of the measurement interval was guided by the principle that time lags in panel studies should correspond to the expected speed of the underlying processes (Dormann and Griffin, 2015). A six-week interval was considered appropriate because repeated exposure to algorithmically curated short-video content can accumulate across ordinary study routines, while attentional control and procrastination can fluctuate over successive assignment and assessment cycles within a semester. This interval was therefore long enough for short-term changes in media-use habits and academic self-regulation to emerge, yet short enough to keep participants within a broadly comparable academic context and to limit attrition and retrospective recall problems. Three waves separated by approximately 6 weeks also permitted the estimation of time-ordered indirect pathways across the academic term while controlling for autoregressive stability and selected reverse paths. Attentional control was conceptualized as a central regulatory pathway linking short-video music consumption to subsequent self-regulatory behavior (academic procrastination) and optimal learning experience (academic flow). By situating music engagement within algorithmically mediated short-video environments, this study contributes to digital media research, music psychology, and educational psychology by clarifying how platform-mediated music exposure may relate to students’ attention regulation and academic experience over time.

On this basis, the following hypotheses were tested.

- H1: short-video music consumption negatively predicts subsequent attentional control.
- H2: attentional control negatively predicts subsequent academic procrastination.
- H3: academic procrastination negatively predicts subsequent academic flow.
- H4: attentional control positively predicts subsequent academic flow.
- H5: short-video music consumption shows prospective indirect associations with academic procrastination and academic flow through attentional control.

## 2 Method

### 2.1 Study design

This study employed a three-wave short-term longitudinal survey design to examine prospective associations among algorithm-driven short-video music consumption, attentional control, academic procrastination, and academic flow among Chinese university students. The study was conducted across one academic term in 2026. The first wave was administered from 5 to 11 January 2026, the second wave from 16 to 22 February 2026, and the third wave from 30 March to 5 April 2026. The interval between adjacent waves was approximately 6 weeks, which was considered appropriate for capturing short-term changes in everyday digital media use and academic self-regulation during an academic term.

### 2.2 Participants and procedure

Participants were recruited from universities in China through online questionnaire distribution. Eligibility criteria required participants to be currently enrolled university students, aged 18 years or above, and regular users of short-video platforms such as Douyin, Kuaishou, TikTok, Xiaohongshu, or Bilibili short videos. Prior to participation, respondents were informed of the study purpose, voluntary participation, confidentiality, and their right to withdraw at any time. A self-generated identification code was used to match responses across the three waves while preserving anonymity. A total of 920 students completed the Time 1 survey. Of these, 870 participated at Time 2 and 820 remained at Time 3. The final retained sample therefore included 820 participants with complete three-wave data. Gender was recorded as male or female. Ethical approval was obtained from Anshan Vocational and Technical College [ID: 202510003].

### 2.3 Measures

#### 2.3.1 Attentional control

Attentional control was measured using the 20-item Attentional Control Scale originally developed by Derryberry and Reed (2002), with Guo et al. (2021) consulted as a subsequent application of the instrument. The scale assesses attentional focusing, resistance to distraction, attentional shifting, and the ability to return attention to an ongoing task after interruption. Participants responded on a four-point scale from 1 (almost never) to 4 (always). After reverse-scoring the relevant items, all 20 items were summed to form a total score ranging from 20 to 80, with higher scores indicating better overall attentional control. In the theoretical interpretation of the study, lower attentional control was understood as reflecting greater susceptibility to attention fragmentation in algorithm-driven short-video environments. Cronbach’s alpha values were satisfactory across the three waves: T1 = 0.884, T2 = 0.879, T3 = 0.880.

#### 2.3.2 Academic procrastination

Academic procrastination was measured using the 25-item Academic Procrastination Scale originally developed by McCloskey (2011), with Tian et al. (2024) consulted as a recent application among university students and translated by Yip and Chung (2022) to Chinese. The items assess unnecessary delay in initiating or completing academic responsibilities, distraction by competing activities, and ineffective management of academic tasks. Responses were given on a five-point Likert scale from 1 (disagree) to 5 (agree); the relevant items were reverse-scored before mean scores were calculated, with higher scores representing greater academic procrastination. Cronbach’s alpha values were high across the three waves: T1 = 0.941, T2 = 0.940, T3 = 0.943.

#### 2.3.3 Academic flow

Academic flow was measured using the 10 core flow items of the Flow Short Scale developed by Rheinberg et al. (2003). The items assess fluency of performance and absorption, including concentration, perceived control, intrinsic involvement, and immersion during academic tasks. Participants responded on a five-point Likert scale, and mean scores were calculated such that higher values indicated stronger academic flow. Cronbach’s alpha values were satisfactory across the three waves: T1 = 0.869, T2 = 0.858, T3 = 0.869.

#### 2.3.4 Short-video music consumption

Short-video music consumption was operationalized as a four-item behavioral exposure index rather than as a reflective psychological scale. The items assessed complementary aspects of exposure to and engagement with music-containing short videos, including how often students encountered such content, attended to it because of the music, continued viewing because of music, and moved to additional algorithmically recommended music-based clips. Participants responded using a five-point frequency scale from 1 (never) to 5 (very often), and the four responses were averaged to form an observed composite score. Because the indicators describe distinct behavioral components that jointly define the exposure index, rather than interchangeable manifestations caused by a single latent trait, EFA or CFA was not treated as the appropriate validation framework. The complete item wording is presented in Supplementary Table S1 for transparency.

### 2.4 Data analysis

Data analyses were conducted in R. First, scores were calculated for each construct at each wave. Academic procrastination and academic flow were represented by mean scores, attentional control by the total score of the 20 attentional-control items, and short-video music consumption by the mean of the four behavioral exposure indicators. The latter was analyzed as an observed composite index because its indicators were specified as complementary components of exposure rather than as reflective indicators of a latent psychological trait. Descriptive statistics and attrition analyses were then conducted. Attrition was evaluated by comparing students retained through Time 3 with those who dropped out after earlier waves on Time 1 study variables and baseline short-video use.

Pearson correlations were calculated to examine bivariate associations among the four core variables across the three waves. A cross-lagged panel model was then estimated using observed scale scores. The model included autoregressive paths for each construct, hypothesized cross-lagged paths from short-video music consumption to later attentional control, from attentional control to later academic procrastination and academic flow, and from academic procrastination to later academic flow. Reverse paths were also included to provide a more conservative test of directionality. Missing data were handled using full-information maximum likelihood. Bootstrapping with 5,000 resamples was used to estimate confidence intervals for indirect effects. Model fit was evaluated using the comparative fit index (CFI), Tucker-Lewis index (TLI), root mean square error of approximation (RMSEA), and standardized root mean square residual (SRMR).

## 3 Results

### 3.1 Sample characteristics, attrition, and descriptive statistics

Table 1 summarizes the sample characteristics, attrition pattern, and descriptive statistics across the three waves. A total of 920 students completed the first survey, 870 completed the second survey, and 820 completed the third survey. Gender distribution was balanced across the three waves. At Time 1, 50.76% of participants were male and 49.24% were female. Douyin was the most frequently reported short-video platform at all three waves, followed by Xiaohongshu, Kuaishou, Bilibili short videos, TikTok, and other platforms.

**Table 1:** Sample characteristics, attrition analysis, and descriptive statistics across three waves.

Variable	Time 1	Time 2	Time 3	Attrition *p*
*N*	920	870	820	
Gender: Male	467 (50.76%)	443 (50.92%)	413 (50.37%)	
Gender: Female	453 (49.24%)	427 (49.08%)	407 (49.63%)	
Year 1	211 (22.93%)	199 (22.87%)	183 (22.32%)	
Year 2	241 (26.20%)	228 (26.21%)	218 (26.59%)	
Year 3	241 (26.20%)	229 (26.32%)	215 (26.22%)	
Year 4	227 (24.67%)	214 (24.60%)	204 (24.88%)	
Main platform: Douyin	418 (45.43%)	400 (45.98%)	381 (46.46%)	
Main platform: Xiaohongshu	158 (17.17%)	147 (16.90%)	139 (16.95%)	
Main platform: Kuaishou	126 (13.70%)	119 (13.68%)	110 (13.41%)	
Main platform: Bilibili short videos	105 (11.41%)	99 (11.38%)	94 (11.46%)	
Main platform: TikTok	47 (5.11%)	44 (5.06%)	39 (4.76%)	
Main platform: Other	66 (7.17%)	61 (7.01%)	57 (6.95%)	
Daily short-video use, M (SD)	2.12 (0.86)	2.25 (0.84)	2.41 (0.85)	0.691
Short-video music consumption, M (SD)	3.17 (0.61)	3.22 (0.61)	3.33 (0.63)	0.775
Attentional control, M (SD)	54.29 (7.47)	53.95 (7.41)	53.15 (7.44)	0.469
Academic procrastination, M (SD)	2.81 (0.51)	2.87 (0.50)	2.96 (0.52)	0.273
Academic flow, M (SD)	3.40 (0.54)	3.31 (0.52)	3.27 (0.54)	0.719

Attrition p values compare participants retained through Time 3 with those who dropped out after Time 1 or Time 2 on baseline variables. Attentional control is scored as a total score ranging from 20 to 80, with higher scores indicating better attentional control.

**Figure 1:**
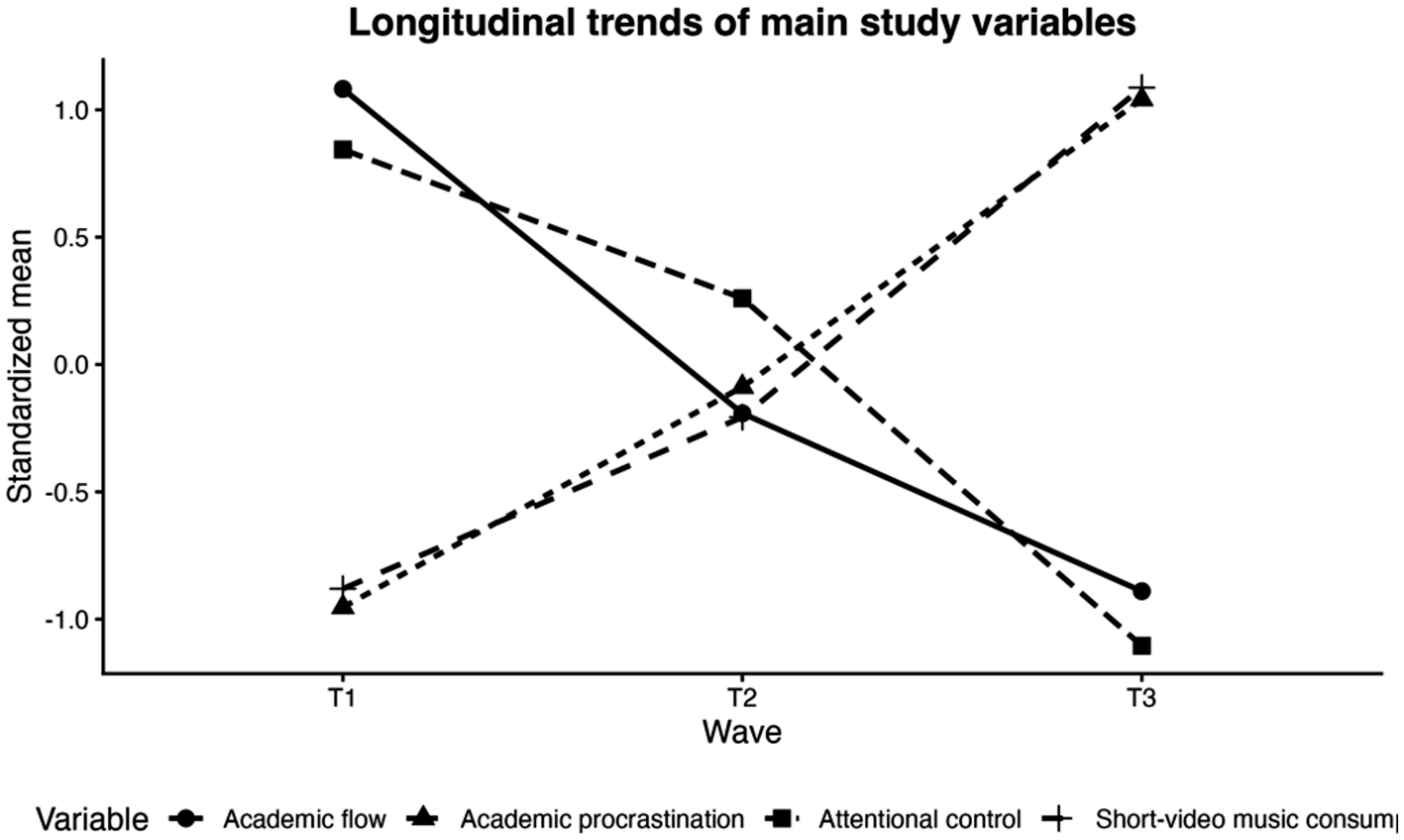
Longitudinal trends of short-video music consumption, attentional control, academic procrastination, and academic flow across three waves.

Attrition analyses indicated that students who remained through Time 3 did not differ significantly from those who dropped out on baseline short-video music consumption, attentional control, academic procrastination, academic flow, or daily short-video use. This suggests that attrition bias was not substantial. Descriptive trends showed that short-video music consumption increased from Time 1 to Time 3, while attentional control gradually decreased. Academic procrastination showed a gradual increase, whereas academic flow showed a gradual decrease across the three waves (Figure 1).

### 3.2 Model fit

The observed-variable cross-lagged panel model demonstrated acceptable to good fit to the data: CFI = 0.991, TLI = 0.963, RMSEA = 0.060, and SRMR = 0.013. The robustness model that additionally controlled for baseline daily short-video use produced CFI = 0.970, TLI = 0.909, RMSEA = 0.086, and SRMR = 0.074. Relative to the primary model, the lower TLI and elevated RMSEA indicate weaker and only marginal absolute fit rather than equally good fit. This modest deterioration is plausible because adding baseline daily short-video use introduces additional covariance to be reproduced while the sensitivity model retains a parsimonious path structure; associations involving the control that are not explicitly modeled can therefore appear as residual misfit. Nevertheless, the CFI and SRMR remained broadly compatible with tolerable fit under conventional joint-index guidance (Hu and Bentler, 1999), and the substantive path estimates remained stable. The robustness model was therefore interpreted cautiously as a sensitivity analysis supporting the stability of the main pattern, not as a replacement for the better-fitting primary model (Table 2).

**Table 2:** Model fit indices.

Model	*χ* ^2^	df	*p*	CFI	TLI	RMSEA	SRMR
Primary CLPM	68.437	16	<0.001	0.991	0.963	0.060	0.013
Robustness model with baseline daily short-video use	202.736	26	<0.001	0.970	0.909	0.086	0.074

### 3.3 Correlations among study variables across waves

Table 3 presents the zero-order correlations among the four main variables across the three waves. The correlations were in the expected directions. Short-video music consumption was negatively correlated with attentional control and academic flow, and positively correlated with academic procrastination. Attentional control was negatively correlated with academic procrastination and positively correlated with academic flow. Academic procrastination was negatively correlated with academic flow.

**Table 3:** Correlations among main variables across three waves.

Variable	SVM_T1	AC_T1	AP_T1	AF_T1	SVM_T2	AC_T2	AP_T2	AF_T2	SVM_T3	AC_T3	AP_T3	AF_T3
SVM_T1	1.000	−0.286	0.422	−0.425	0.553	−0.394	0.398	−0.378	0.391	−0.368	0.375	−0.373
AC_T1	−0.286	1.000	−0.439	0.530	−0.253	0.617	−0.508	0.500	−0.244	0.406	−0.440	0.464
AP_T1	0.422	−0.439	1.000	−0.633	0.403	−0.440	0.670	−0.563	0.346	−0.392	0.466	−0.480
AF_T1	−0.425	0.530	−0.633	1.000	−0.392	0.492	−0.544	0.658	−0.303	0.439	−0.456	0.505
SVM_T2	0.553	−0.253	0.403	−0.392	1.000	−0.350	0.382	−0.354	0.625	−0.386	0.368	−0.366
AC_T2	−0.394	0.617	−0.440	0.492	−0.350	1.000	−0.445	0.458	−0.329	0.726	−0.461	0.480
AP_T2	0.398	−0.508	0.670	−0.544	0.382	−0.445	1.000	−0.489	0.349	−0.414	0.783	−0.498
AF_T2	−0.378	0.500	−0.563	0.658	−0.354	0.458	−0.489	1.000	−0.310	0.420	−0.408	0.717
SVM_T3	0.391	−0.244	0.346	−0.303	0.625	−0.329	0.349	−0.310	1.000	−0.367	0.364	−0.350
AC_T3	−0.368	0.406	−0.392	0.439	−0.386	0.726	−0.414	0.420	−0.367	1.000	−0.434	0.426
AP_T3	0.375	−0.440	0.466	−0.456	0.368	−0.461	0.783	−0.408	0.364	−0.434	1.000	−0.439
AF_T3	−0.373	0.464	−0.480	0.505	−0.366	0.480	−0.498	0.717	−0.350	0.426	−0.439	1.000

SVM = short-video music consumption; AC = attentional control; AP = academic procrastination; AF = academic flow. All correlations were significant at *p* < 0.001.

### 3.4 Cross-lagged associations

**Figure 2:**
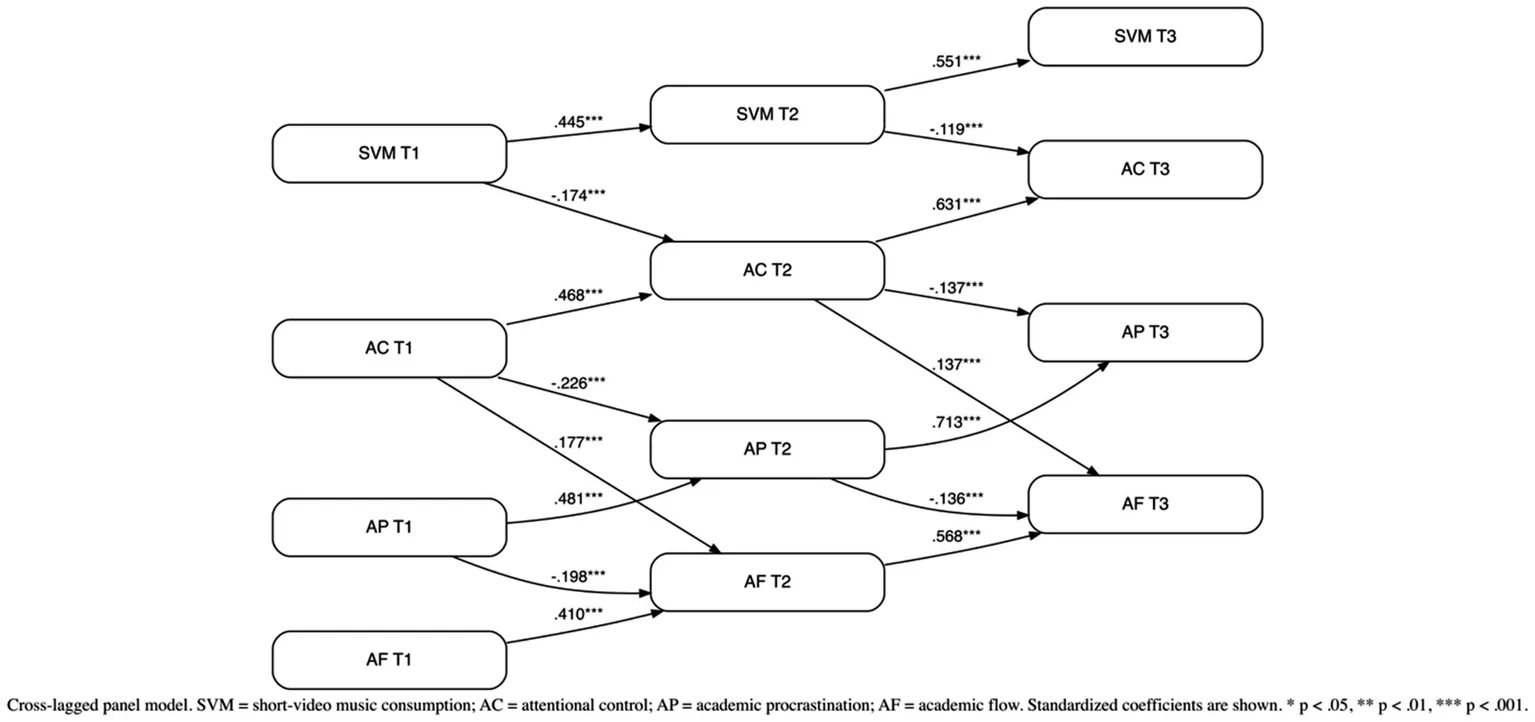
Cross-lagged panel model linking short-video music consumption, attentional control, academic procrastination, and academic flow.

Table 4 reports the standardized path coefficients for the autoregressive paths and the hypothesized cross-lagged paths. All autoregressive paths were significant, indicating moderate to strong stability in short-video music consumption, attentional control, academic procrastination, and academic flow across adjacent waves. Higher short-video music consumption predicted lower subsequent attentional control from Time 1 to Time 2 and from Time 2 to Time 3. Better attentional control predicted lower subsequent academic procrastination and higher subsequent academic flow. Academic procrastination predicted lower subsequent academic flow across both adjacent intervals. These findings support the proposed longitudinal pattern linking short-video music consumption, attentional control, procrastination, and academic flow. To examine whether the observed associations were strictly unidirectional, reverse cross-lagged paths were also estimated. The full set of reverse paths is reported in Supplementary Table S1. Several reverse paths reached statistical significance, suggesting reciprocal dynamics between short-video music consumption and academic self-regulation, although the hypothesized forward paths remained significant after accounting for these reverse associations (Figure 2).

**Table 4:** Autoregressive and cross-lagged path coefficients.

Path type	Path	*B*	SE	95% CI	*β*	*p*
Autoregressive	SVM_T1 → SVM_T2	0.445	0.032	[0.383, 0.506]	0.445	<0.001
Autoregressive	SVM_T2 → SVM_T3	0.562	0.032	[0.500, 0.627]	0.551	<0.001
Autoregressive	AC_T1 → AC_T2	0.466	0.028	[0.411, 0.521]	0.468	<0.001
Autoregressive	AC_T2 → AC_T3	0.633	0.028	[0.580, 0.690]	0.631	<0.001
Autoregressive	AP_T1 → AP_T2	0.481	0.031	[0.420, 0.542]	0.481	<0.001
Autoregressive	AP_T2 → AP_T3	0.731	0.027	[0.678, 0.782]	0.713	<0.001
Autoregressive	AF_T1 → AF_T2	0.400	0.032	[0.337, 0.465]	0.410	<0.001
Autoregressive	AF_T2 → AF_T3	0.592	0.029	[0.536, 0.649]	0.568	<0.001
Hypothesized	SVM_T1 → AC_T2	−2.109	0.340	[−2.770, −1.436]	−0.174	<0.001
Hypothesized	SVM_T2 → AC_T3	−1.442	0.308	[−2.040, −0.838]	−0.119	<0.001
Hypothesized	AC_T1 → AP_T2	−0.015	0.002	[−0.019, −0.012]	−0.226	<0.001
Hypothesized	AC_T2 → AP_T3	−0.010	0.002	[−0.013, −0.006]	−0.137	<0.001
Hypothesized	AP_T1 → AF_T2	−0.204	0.034	[−0.270, −0.137]	−0.198	<0.001
Hypothesized	AP_T2 → AF_T3	−0.146	0.030	[−0.205, −0.088]	−0.136	<0.001
Hypothesized	AC_T1 → AF_T2	0.012	0.002	[0.009, 0.016]	0.177	<0.001
Hypothesized	AC_T2 → AF_T3	0.010	0.002	[0.006, 0.014]	0.137	<0.001

### 3.5 Longitudinal indirect effects and robustness checks

Bootstrapped indirect effects are shown in Table 5. Short-video music consumption showed a significant positive indirect association with later academic procrastination through lower attentional control. Because attentional control was scored such that higher values indicated better control, the positive indirect effect indicates that higher short-video music consumption was associated with reduced attentional control, which in turn was associated with greater academic procrastination. Short-video music consumption also showed a significant negative indirect association with academic flow through attentional control, indicating that higher short-video music consumption was prospectively linked to lower academic flow through reduced attentional control. In addition, attentional control showed a significant indirect association with academic flow through academic procrastination.

**Table 5:** Bootstrapped longitudinal indirect effects.

Indirect pathway	*B*	SE	95% CI	*β*	*p*
SVM_T1 → AC_T2 → AP_T3	0.020	0.005	[0.011, 0.031]	0.024	<0.001
SVM_T1 → AC_T2 → AF_T3	−0.021	0.005	[−0.032, −0.011]	−0.024	<0.001
AC_T1 → AP_T2 → AF_T3	0.002	0.001	[0.001, 0.003]	0.031	<0.001

SVM = short-video music consumption; AC = attentional control; AP = academic procrastination; AF = academic flow. *B* values are unstandardized indirect effects, *β* values are standardized indirect effects, and confidence intervals refer to the unstandardized estimates based on 5,000 bootstrap resamples.

The robustness model controlling for baseline daily short-video use showed that the main path pattern remained consistent. Specifically, short-video music consumption continued to predict lower subsequent attentional control, attentional control continued to predict lower subsequent academic procrastination and higher academic flow, and academic procrastination continued to predict lower academic flow. The model’s CFI and SRMR remained within broadly tolerable ranges, whereas its TLI and RMSEA indicated weaker, marginal fit. Accordingly, the robustness findings were treated as supplementary evidence that the principal coefficients were not explained solely by baseline viewing duration, while the primary model remained the basis for substantive interpretation.

## 4 Discussion

### 4.1 Summary of Main findings

The present three-wave longitudinal study examined prospective associations among algorithm-driven short-video music consumption, attentional control, academic procrastination, and academic flow in a sample of Chinese university students. Across two cross-lagged intervals, higher short-video music consumption prospectively predicted lower subsequent attentional control; better attentional control predicted both lower subsequent academic procrastination and higher subsequent academic flow; and higher academic procrastination predicted lower subsequent academic flow. Bootstrapped indirect effects further indicated that attentional control served as a longitudinal pathway linking short-video music consumption to later academic procrastination and academic flow, and that academic procrastination linked attentional control to academic flow. These patterns held after accounting for autoregressive stability across the three waves, and the main path structure remained consistent in a robustness model that additionally adjusted for baseline daily short-video use. Taken together, the findings provide longitudinal support for a coherent regulatory chain in which platform-mediated music engagement is prospectively associated with attentional and motivational outcomes that shape students’ academic experience (Xie et al., 2023; Ye et al., 2025; Liu and Li, 2024).

### 4.2 Short-video music consumption and attentional control

The negative prospective association between short-video music consumption and subsequent attentional control should be interpreted with care. The present findings do not imply that music itself, as a class of stimulus, weakens attention (Schäfer et al., 2013; Cheah et al., 2022; De Francesco et al., 2025). Rather, they suggest that music consumed within algorithm-driven short-video environments—characterized by rapid clip succession, emotional intensification, and platform-curated trends—may contribute to a more fragmented attentional environment over time (Yan et al., 2024; Ye et al., 2025; Chen et al., 2023b). Repeated exposure to such material may gradually entrench patterns of rapid attentional switching and external-cue dependence, both of which are inconsistent with the sustained focus and active disengagement from distractors that attentional control requires (Ophir et al., 2009; Parry and le Roux, 2021; Petersen and Posner, 2012). The longitudinal pattern is therefore consistent with the conceptualization of attention fragmentation outlined above, in which the measured decline in attentional control reflects greater susceptibility to such fragmentation. Given the observational design, this association is framed as a prospective relationship rather than as evidence that short-video music consumption causally diminishes attentional capacity (Hamaker et al., 2015; Lucas, 2023).

The reverse paths further caution against a purely one-directional interpretation. Several reverse associations were statistically significant, indicating that students with lower attentional control or weaker academic self-regulation may also be more likely to engage with algorithmically curated short-video content over time. One plausible explanation is selection: students who find sustained concentration effortful may be especially drawn to rapidly changing, immediately rewarding content that requires little commitment and continually supplies novel cues. A second possibility is reciprocal reinforcement, whereby initially lower attentional control increases short-video engagement and repeated engagement subsequently makes sustained academic attention more difficult. The present CLPM cannot determine whether one process predominates, but the persistence of the hypothesized forward paths after reverse paths were included suggests that the findings are not reducible to a single reverse-causality account.

The findings also should not be interpreted as isolating a purely auditory music effect. On short-video platforms, music is normally synchronized with visual cuts, movement, captions, social cues, and emotionally salient narratives. Music may increase arousal, memorability, and anticipation, while visual editing and algorithmic sequencing simultaneously guide orienting and repeated viewing. The measured exposure therefore represents an inherently audio-visual platform experience: the observed associations may arise from music, visual stimulation, or their interaction. This qualification narrows the claim of the study from ‘music impairs attention’ to the more specific proposition that music-embedded, algorithmically curated short-video consumption is prospectively associated with attentional control. Experimental studies comparing audio-only, visual-only, and combined audio-visual conditions are needed to identify the contribution of each component.

### 4.3 Attentional control and academic procrastination

The finding that better attentional control predicted lower subsequent academic procrastination supports the view that procrastination is, in part, an attention-regulation problem rather than solely a motivational or time-management failure (Steel, 2007; Steel and Klingsieck, 2016; Xie et al., 2023). Students with stronger attentional control may be better positioned to initiate effortful academic tasks, resist competing digital cues, and recover from interruptions during sustained study (Hofmann et al., 2012; Eysenck et al., 2007). By contrast, lower attentional control may render academic tasks subjectively more effortful and aversive, particularly when more immediately rewarding alternatives such as short-video content remain easily accessible, thereby increasing the likelihood of voluntary delay (Tice and Bratslavsky, 2000; Sirois and Pychyl, 2013; Liu et al., 2022). This interpretation aligns with contemporary self-regulation perspectives that locate procrastination at the interface of attention, emotion regulation, and task aversiveness (Baumeister and Heatherton, 1996; Sirois and Pychyl, 2013), and extends them by providing longitudinal evidence that variation in attentional control prospectively shapes procrastination tendencies across an academic term.

### 4.4 Attentional control, academic procrastination, and academic flow

Academic procrastination predicted lower subsequent academic flow, and attentional control predicted academic flow both indirectly via procrastination and directly. These results are theoretically coherent. Flow requires sustained involvement, continuous task engagement, and a balance between challenge and perceived skill (Csikszentmihalyi, 1990; Shernoff et al., 2003; Engeser and Rheinberg, 2008); procrastination disrupts these conditions by delaying task initiation, generating time pressure and negative affect, and fragmenting the work process into short and effortful episodes (Steel, 2007; Sirois and Pychyl, 2013). The presence of a direct path from attentional control to academic flow further suggests that attention regulation contributes to flow beyond its role in preventing procrastination: stronger attentional control may directly enable the deep absorption, resistance to interruption, and continuous immersion that flow entails (Derryberry and Reed, 2002; Petersen and Posner, 2012). Together, these results indicate that academic flow depends both on the prior allocation of effort and on the in-task capacity to maintain immersion, which attentional control supports.

### 4.5 Longitudinal indirect effects and the overall regulatory pathway

The bootstrapped indirect effects converged on a coherent regulatory pathway: higher short-video music consumption was associated with reduced subsequent attentional control, which was in turn associated with greater academic procrastination and lower academic flow; and attentional control was further linked to academic flow through procrastination. Interpreting these effects requires attention to scoring direction. Because higher attentional control scores reflect better control, the positive indirect effect of short-video music consumption on academic procrastination via attentional control indicates that higher consumption is prospectively linked to lower attentional control, which in turn is linked to higher procrastination (Xie et al., 2023; Ye et al., 2025). Analogously, the negative indirect effect on academic flow indicates that higher consumption is prospectively linked to lower flow through reduced attentional control. The convergence of these pathways suggests that attentional control is a central psychological pathway connecting platform-mediated music engagement with downstream self-regulation and learning experience (Yan et al., 2024; Chen et al., 2023b). These associations are characterized as prospective indirect pathways and time-ordered associations rather than as evidence of causal mediation, given the observational nature of the design and the possibility of unmeasured confounding (Hamaker et al., 2015; Lucas, 2023).

### 4.6 Theoretical contributions

The present findings make three theoretical contributions. First, they extend digital media research by identifying a music-mediated mechanism through which short-video platforms may relate to students’ attention and academic functioning. Whereas prior work has focused largely on screen time, problematic smartphone use, and social media addiction, the present results highlight short-video music consumption as a distinct and measurable behavioral pattern with prospective links to attentional control and academic outcomes. Second, the study contributes to music psychology by examining music engagement within algorithmically curated short-video environments rather than within the traditional contexts of intentional, continuous, and self-selected listening typically assumed in the field. This reframing suggests that the psychological functions of music may operate differently when music is delivered through fragmented, retention-oriented digital platforms. Third, the study contributes to educational psychology by linking platform-mediated music exposure to a sequence of regulatory and experiential outcomes through a three-wave longitudinal design, thereby moving beyond the predominantly cross-sectional evidence base in this area and clarifying the temporal ordering among constructs that have often been studied in isolation.

### 4.7 Practical implications

The findings carry practical implications at three levels. For students, the implication is not that music engagement should be avoided, but that intentional, self-selected music listening should be distinguished from algorithm-driven short-video music consumption, the latter of which may be more closely tied to attention fragmentation in everyday learning contexts. For universities, algorithmic media literacy could be incorporated into first-year orientation, study-skills courses, academic advising, and learning-support programs. Rather than focusing only on total screen time, these services could help students recognize how recommendation systems, repeated music cues, autoplay, and audio-visual salience shape attention and prolong platform use. Academic counselors could support students in reviewing their own usage patterns, creating platform-free study periods, disabling autoplay or nonessential notifications, and separating purposeful music listening from open-ended scrolling. Mental-health and counseling services should avoid treating frequent use as a diagnosis, but may consider it alongside procrastination, concentration difficulties, and reduced academic flow when discussing students’ self-regulatory routines. At the institutional level, digital-wellbeing campaigns and campus learning platforms could provide evidence-based guidance on intentional media use without moralizing or stigmatizing students’ leisure practices.

## 5 Limitations

Several limitations should be acknowledged. First, although the three-wave design establishes temporal ordering more effectively than a cross-sectional design, the study remains observational and does not establish causality. Unmeasured time-varying confounders may contribute to the observed associations. Second, the traditional CLPM combines stable between-person differences with within-person fluctuations. Consequently, a cross-lagged coefficient may partly reflect that students who are generally heavier users also differ stably in attentional control, rather than showing that an increase in one student’s use predicts a subsequent within-person change. Future studies should use random-intercept cross-lagged panel models, latent curve models with structured residuals, or intensive longitudinal designs to separate these sources of variation (Hamaker et al., 2015; Lucas, 2023). Third, all constructs were assessed by self-report, creating potential common method variance, recall error, and socially desirable responding. The temporal separation of the three waves and the inclusion of autoregressive and reverse paths reduce some same-occasion inflation, but they do not eliminate shared-method bias (Podsakoff et al., 2003). Harman’s single-factor test was not treated as decisive evidence because failure to extract one dominant factor cannot rule out method effects; future research should combine self-reports with passive platform logs, screen-time records, behavioral attention tasks, teacher or peer reports, and, where feasible, latent method-factor approaches. Fourth, longitudinal measurement invariance was not formally tested because the present analysis used observed composite scores rather than longitudinal latent measurement models. Without invariance testing, it cannot be confirmed that the meaning and metric of each construct were fully equivalent across waves. Future item-level studies should test configural, metric, and scalar invariance before interpreting latent mean or within-person changes. Fifth, the four-item short-video music measure was designed as a brief behavioral exposure index rather than a validated reflective psychological scale. Although this operationalization suits the descriptive frequency-based indicators used here, future work should independently replicate its content coverage and examine convergent validity against objective platform-use data. Sixth, short-video music is inherently embedded in visual and algorithmic content; the study cannot isolate the effects of music from visual editing, narrative content, social cues, or their interactions. Seventh, the sample consisted exclusively of Chinese university students, so generalization to other cultures, age groups, platforms, and educational systems should be made cautiously. Finally, the approximately six-week intervals capture short-term change within one academic term but cannot address longer-term developmental trajectories or effects that unfold over days rather than weeks.

## 6 Conclusion

In the digital platform era, music has become not only an aesthetic and emotional resource but also a structural element of algorithmically curated attention environments. Drawing on three waves of longitudinal data from Chinese university students, the present study indicates that music-embedded short-video consumption is prospectively associated with lower attentional control, greater academic procrastination, and reduced academic flow, with attentional control functioning as a key time-ordered regulatory pathway. These findings do not establish that music alone causes academic difficulties; rather, they highlight the importance of examining music within the fragmented, audio-visual, and retention-oriented architecture of short-video platforms. Higher education institutions should incorporate algorithmic media literacy into academic counseling, study-skills provision, and student-support services by helping students understand recommendation mechanisms, identify attention-capturing design features, monitor their own use, and establish intentional media boundaries during academic work. Future research should use RI-CLPM and other within-person longitudinal models, objective platform and attention measures, experimental comparisons of audio-only, visual-only, and combined stimuli, and cross-cultural samples to test the generality and mechanisms of these associations.

## Data Availability

The raw data supporting the conclusions of this article will be made available by the authors, without undue reservation.
