# Supplementary material for: Algorithmic short-video music consumption and academic related functions: a three-wave longitudinal study of university students
Source: Front Psychol. 2026 Aug 27;17:1907189. doi: 10.3389/fpsyg.2026.1907189 (PMC13558422; doi:10.3389/fpsyg.2026.1907189)
Supplement: Supplementary file 1 [file Table_1.docx]

**Table S1. Items of the short-video music consumption**

| **Item** | **Item wording** | **Response format** |
| --- | --- | --- |
| 1 | How often do you encounter short videos in which music or a song is a prominent part of the content? | 1 = Never to 5 = Very often |
| 2 | How often does the background music or song make you pay more attention to a short video? | 1 = Never to 5 = Very often |
| 3 | How often do you continue watching short videos longer than intended because of the music or song used in them? | 1 = Never to 5 = Very often |
| 4 | How often do music-based recommendations lead you to watch additional short videos using the same or similar songs? | 1 = Never to 5 = Very often |
